# Supplementary material for: Mortality Prediction of Patients with Subarachnoid Hemorrhage Using a Deep Learning Model Based on an Initial Brain CT Scan
Source: Brain Sci. 2023 Dec 22;14(1):10. doi: 10.3390/brainsci14010010 (PMC10812955; doi:10.3390/brainsci14010010)
Supplement: Supplementary file 1 [file brainsci-14-00010-s001.zip › brainsci-2730127-supplementary.pdf]

SUPLEMENTARY MATERIALS

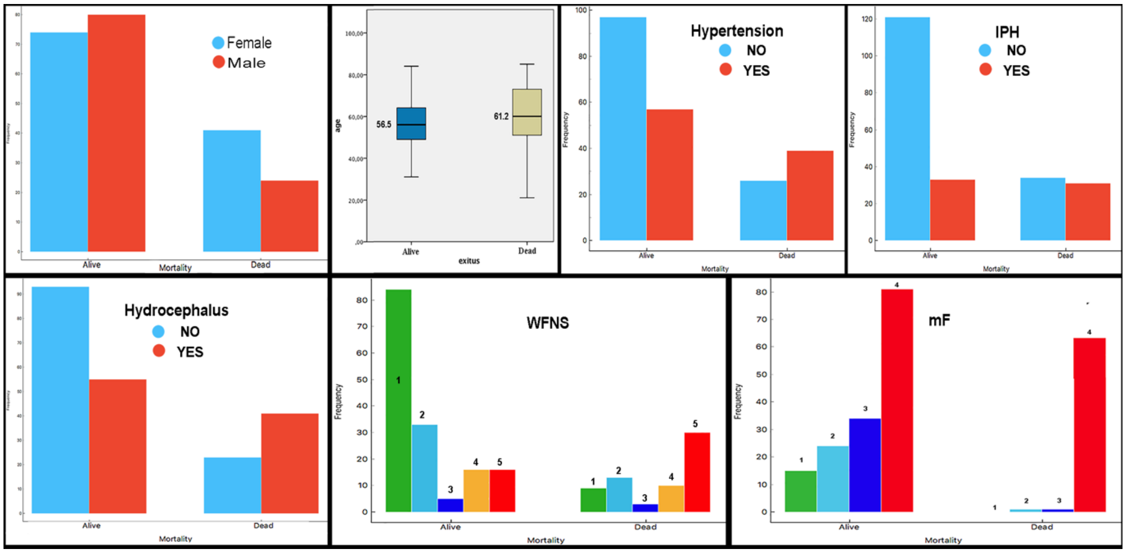

Supplementary Figure S1. Distribution of mortality according to Risk factors: **A**: Sex; **B**: Age; **C**: Hypertension (HT); **D**: Intraparenchymal hematoma (IPH); **E**: Acute Hydrocephalus; **F**: WFNS and **G**: Modified Fisher (mF).

Supplementary Table S1. CT scan protocol parameters. *CTDIvol: Computed Tomography Dose Index volume.*

Manufacturer	PHILIPS	SIEMENS
Model	Ingenuity CT	Somatom
Scan Mode	Spiral	Spiral
Tube Voltage (kV)	120	120
Exposure(mAs)	189-221	199-262
Average CTDIvol (mGy)	48	57
Rotation Time (s)	0.4	1
Collimation	64x 0.625	64x0.625
Pitch factor	0.3	0.5
Slice Thickness(mm)	3-4	2-4
Number of slices	64-99	66-150
Field of view (mm)	250	250
Samples per pixel	1	1
CT detector array	512x512	512x512

Supplementary Table S2. Performance of Neural Networks Algorithms. Results for classes. FDR: False Discovery Rate; FN: False Negative; FP: False Positive; R: Rate; TN: True Negative; TP: True Positive.

		**Image-based Neural Network performance**				**Image and Metadata based Neural Network performance**			
**Class**	**Metric**	Best AUC	Best F1	Best Loss	Last	Best AUC	Best F1	Best Loss	Last
**ALIVE**	**TP**	27	25	19	22	29	24	23	30
	**TN**	2	5	11	10	3	9	8	0
	**FP**	11	8	2	3	10	4	5	13
	**FN**	3	5	11	8	1	6	7	0
	**Sensitivity**	0.90	0.83	0.63	0.73	0.97	0.80	0.77	1
	**Specificity**	0.15	0.38	0.85	0.77	0.23	0.69	0.62	0
	**Precision**	0.71	0.76	0.90	0.88	0.74	0.86	0.82	0.70
	**FPR**	0.85	0.62	0.15	0.23	0.77	0.31	0.38	1
	**FNR**	0.10	0.17	0.37	0.27	0.03	0.20	0.23	0.00
	**FDR**	0.29	0.24	0.10	0.12	0.26	0.14	0.18	0.30
	**Accuracy**	0.67	0.70	0.70	0.74	0.74	0.77	0.72	0.70
	**F1**	0.79	0.79	0.75	0.80	0.84	0.83	0.79	0.82
	**AUC**	0.72	0.74	0.73	0.82	0.78	0.80	0.78	0.35
**DEAD**	**TP**	2	5	11	10	3	9	8	0
	**TN**	27	25	19	22	29	24	23	30
	**FP**	3	5	11	8	1	6	7	0
	**FN**	11	8	2	3	10	4	5	13
	**Sensitivity**	0.15	0.38	0.85	0.77	0.23	0.69	0.62	0
	**Specificity**	0.90	0.83	0.63	0.73	0.97	0.80	0.77	1.00
	**Precision**	0.40	0.50	0.50	0.56	0.75	0.60	0.53	-
	**FPR**	0.10	0.17	0.37	0.27	0.03	0.20	0.23	0
	**FNR**	0.85	0.62	0.15	0.23	0.77	0.31	0.38	1
	**FDR**	0.60	0.50	0.50	0.44	0.25	0.40	0.47	-
	**Accuracy**	0.67	0.70	0.70	0.74	0.74	0.77	0.72	0.70
	**F1**	0.22	0.43	0.63	0.65	0.35	0.64	0.57	0
	**AUC**	0.72	0.74	0.73	0.82	0.78	0.80	0.78	0.35
